# Establishment of Fluorescence Sensitization Method for Hydroxysafflor Yellow A

**DOI:** 10.1155/2020/3027843

**Published:** 2020-04-27

**Authors:** CAO Haiyan, QIN Xiude, LIU Chen, ZHAO Xinzhe, MA Yuhui, ZHOU Jingna, LIU Yu, GE Shaoqin, ZHANG Guowei, NIE Hailiang

**Affiliations:** ^1^College of Traditional Chinese Medicine, Hebei University, Baoding 071000, China; ^2^Shenzhen Hospital of Traditional Chinese Medicine/Fourth Clinical Medical College of Guangzhou University of Traditional Chinese Medicine, Shenzhen 518000, China; ^3^College of Public Health, Hebei University, Baoding 071000, China

## Abstract

As the main active ingredient in Chinese medicine safflower, hydroxysafflor yellow A (HSYA) has multiple pharmacological effects. In the work, the absorption and fluorescence spectra of HSYA under different environmental conditions (such as acidity, temperature, ions, viscosity, and surfactant) were investigated. The fluorescence intensity of HSYA varied greatly with acidity, temperature, viscosity, and surfactant, but was less affected by common cations and anions. Among various surfactants, we found that borax can significantly enhance the HSYA fluorescence intensity, and thus, a borax-HSYA sensitization system for HSYA fluorescence was established. In the optimized sensitization system, the fluorescence intensity of HSYA increased by 20 times and showed a good linearity with HSYA concentrations in the range of 0∼10 *μ*M with a detection limit of 8 nM. The borax-HSYA sensitization system is nontoxic to T24 cells and mice and can be used for the fluorescence imaging of HSYA in cells, thereby providing an effective method for analyzing HSYA *in vitro* and monitoring its metabolism in cells.

## 1. Introduction

Hydroxysafflor yellow A (HSYA), a chalcone glycoside compound, is the main active ingredient in traditional Chinese medicine of safflower. HSYA has multiple pharmacological effects such as analgesic, anti-inflammatory, antifatigue, antihypoxia, and hypotension [1]. It also has broad prospects in the clinical treatment of cardiovascular and cerebrovascular diseases. Therefore, the establishment of analysis method for HSYA is of great significance for studying its pharmacology. So far, high-performance liquid chromatography (HPLC) is the main method for analyzing HSYA. Compared with HPLC, fluorescence analysis has many advantages such as low cost, convenient operation, fast speed, and high sensitivity. Most importantly, in situ imaging and detection of HSYA in biological systems can be achieved with the help of fluorescence microscopy. However, HSYA emits very weak fluorescence in aqueous solution due to the lack of rigid planar configuration in the molecular structure. It is necessary to construct the sensitization system of HSYA to achieve its fluorescence analysis.

In this work, the absorption and fluorescence spectra of HSYA in various microenvironments (such as acidity, temperature, ions, viscosity, and surfactant) were measured. The fluorescence intensity of HSYA varied greatly with acidity, temperature, viscosity, and surfactant, but was less affected by ions. Most importantly, based on our finding that borax can significantly enhance HSYA fluorescence intensity, a borax-HSYA sensitization system for HSYA fluorescence was established. In the sensitization system, the fluorescence intensity of HSYA increased by 20 times, as well as showing a good linear relationship with HSYA concentrations in the range of 0∼10 *μ*M with a low detection limit of 8 nM. In addition, the borax-HSYA sensitization system was nontoxic to T24 cells and mice and can be used for fluorescence imaging of HSYA in cells. Our work will provide an effective method for the analysis of HSYA *in vitro* and its metabolism *in vivo*.

## 2. Experimental Section

### 2.1. Chemicals and Instruments

The chemicals used in the experiment are as follows: HSYA (98.0%, McLean), obtained structure characterization data of the mass spectrum and NMR spectrum, safflower yellow for injection (85.0%, Yongning, Zhejiang), borax (Na_2_B_4_O_7_, 95.5%, McLean), CTAB (99.0%, McLean), *β*-CD (98.0%, McLean), Anti-fluorescence Quenching Sealing Tablets (Biyuntian). The other reagents are commonly used in the laboratory.

The following instruments were used: Fluorescence Spectrophotometer (F-7000, Hitachi, Japan), Ultraviolet-Visible Spectrophotometer (18 Series, Beijing General Purpose), Enzyme labeling instrument (ELx800, Botten Instrument, USA), and Fluorescence microscopy (H550S, Nikon instrument). The other instruments are commonly used in experiments.

### 2.2. Procedure for Spectral Measurement

The stock solution of HSYA (1 mM) and PBS was prepared in ultrapure water and stored at 4°C. Stock solutions of other species such as Na^+^(1 mM), K^+^(1 mM), Mg^2+^(1 mM), Ca^2+^(1 mM), Fe^2+^(1 mM), Fe^3+^(1 mM), Cl^−^(1 mM), CO_3_^2−^(1 mM), CH_3_COO^−^(1 mM), PO_4_^3-^ (1 mM), NO_3_^−^(1 mM), SO_3_^2−^(1 mM), and SO_4_^2−^(1 mM) were freshly prepared in ultrapure water. Finally, the fluorescence spectra of the above solution were measured within the wavelength range of 465 nm to 750 nm in quartz at an excitation wavelength of 450 nm. The fluorescence properties of HSYA under normal conditions were determined. The effects of pH, anion and cation, viscosity, temperature, surfactant, and borax on the spectral properties of HSYA were determined.

### 2.3. Sensitization System of Borax-HSYA

Combined with the experimental results of spectral properties of HSYA, under neutral conditions, borax solution has good water solubility, and borax can enhance the fluorescence intensity of HSYA to a certain extent. Therefore, a borax-HSYA sensitization system can be established to determine the effect of borax solution concentration on the fluorescence intensity of HSYA, and a standard curve can be drawn up. At the same time, the fluorescence intensity and stability of HSYA under the combined influence of temperature and light were measured, and the conditions affecting the stability of HSYA were speculated, which provided a reference for the establishment of a highly sensitive fluorescence sensitization analysis method for HSYA.

### 2.4. Cell Viability Assay

T24 cell was cultured in McCoy's 5A medium (Gibco, USA) and supplemented with 10% fetal bovine serum (Gibco, USA), in a humidified incubator at 37°C with 5% CO_2._

A Cell Counting Kit 8 (CCK-8) was used to evaluate the cytotoxicity of the T24 bladder cancer cells. The 96-well plates were seeded with at a concentration of 5.0 × 10^3^ cells per well. After 12 h, cells were incubated with fresh medium and different concentration drugs (HSYA (0, 10, 25, 50, 100, and 200 *μ*M), borax solution (0, 250, 500, 1000, 2000, and 4000 *μ*M), borax-HSYA (50 *μ*M) system (0, 250, 500, 1000, 2000, and 4000 *μ*M)). After 24 h, a CCK-8 cell cytotoxicity assay kit (Bioss, China) was used. The absorbance was measured at a wavelength of 450 nm, and all experiments were repeated three times. Cell survival rate (%) = (OD value of the experimental group − OD value of the blank group)/(OD value of the control group − OD value of the blank group)  × 100%.

### 2.5. The Acute Toxicity Test

Kunming mice were purchased from the Experimental Animal Center of Hebei Province (Shijiazhuang, China), used after 3 d of acclimatization. All animals were handled in accordance with the Principles for Care and Use of Experimental Animals from Hebei University and approved by the Institutional Committee on Animal Care. They were maintained under standard environmental conditions (23 ± 2°C, 55 ± 5% humidity and 12 h/12 h light/dark cycle). All animals were allowed free access to tap water and standard laboratory rat food.

Three groups were designed, borax group (4000 *μ*M) and borax-HSYA group (4000 *μ*M and 7.14 mg/mL) and control group (sterilized saline), 10 mice in each group. The drugs were injected in mice, by tail vein. The dosage of the borax-HSYA group is 0.2 mL/10 g which was 100 times that of human normal intravenous drip. The experimental animals were injected with 3 times the dose of sodium pentobarbital sodium for 30 mg/kg, and the subsequent experiment was carried out after the death was confirmed.

### 2.6. Cell Fluorescence Imaging

Bladder cancer cell line T24 was preinoculated on a 6-well culture plate. The bladder cancer T24 cells were treated with 1% streptomycin and 10% fetal bovine serum. The 6-well plate was placed in a CO_2_ incubator at 37°C and 5% concentration for 24 hours. After cell adherence, the bladder cancer cells were treated with 10 *μ*L HSYA 6 mg/mL, 24 mg/mL of high concentration sensitization system, and PBS buffer solution (control group), respectively. After 12 hours of treatment, the cells were taken out. The plate was washed three times with PBS buffer solution and fixed with room temperature polyformaldehyde solution for 15 minutes. After discarding the fixative solution, PBS was washed three times for 3 minutes each time. The liquid on the surface was dried and dripped with antifluorescence quenching tablets. The cell climbing film was taken by fluorescence microscopy for fluorescence imaging of cells.

### 2.7. Statistical Analysis

SPSS 19.0 statistical software was used for one-way ANOVA test. LSD *t*-test was used for two-way comparison between groups. Measurement data were expressed as mean ± standard deviation ($\bar{x}$ ± *s*). The test level was *α* = 0.05, and ANOVA was performed.

## 3. Results

### 3.1. Spectral Properties of HSYA

#### 3.1.1. Effects of Acidity and Temperature on HSYA Spectra

**Figure 1:**
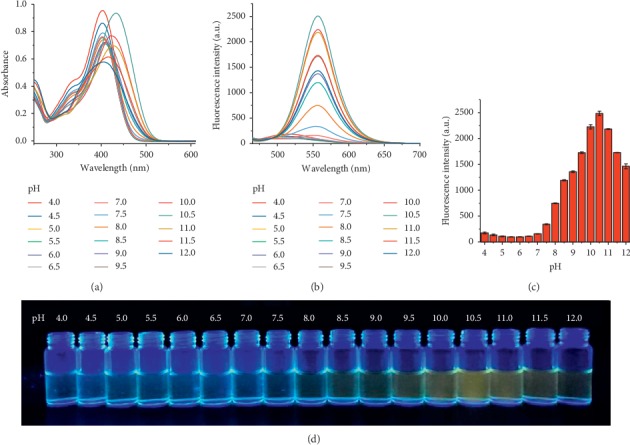
(a) Absorption spectra and (b) fluorescence spectra of HSYA under different acidity conditions (pH 4.0–12.0). All spectra were measured in PBS (PO_4_^3−^ = 10 mM) solution at room temperature, with excitation at 450 nm. (c) Changes in maximum fluorescence intensity of HSYA with increasing pH from 4.0 to 12.0. (d) Fluorescence images of HSYA at various pH under 365 nm ultraviolet lamp.

**Figure 2:**
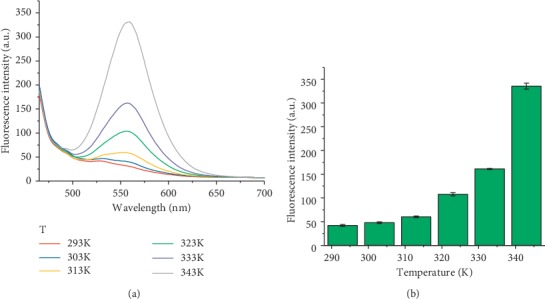
(a) Fluorescence spectra of HSYA at various temperatures (293∼343K). All spectra were measured in PBS (PO_4_^3−^ = 10 mM, pH = 7) solution, with excitation at 450 nm. (b) Changes in maximum fluorescence intensity of HSYA with temperature values (293∼343K).

As shown in Figure 1, HSYA showed maximum absorption around 400 nm under acidic and weak alkaline conditions, accompanying a gradual decrease in absorption intensity with increasing pH from 4.0 to 9.0 (Figure 1(a)). As pH rose further to 11.5, the absorption wavelength underwent a redshift of above 15 nm. In the fluorescence spectra (Figure 1(b)), HSYA displayed an emission in the range of 490∼650 nm with a peak around 555 nm. The fluorescence intensity remained almost stable in acidic environment (pH ≤ 7), gradually enhanced in the pH range of 7.0∼10.5, and stepwise decreased in the pH range of 10.5∼12.0 (Figure 1(c)). The HSYA solution emitted orange fluorescence under ultraviolet (365 nm) irradiation and reached a maximum level at pH 10.5 (Figure 1(d)). Changes in pH-dependent fluorescence images were consistent with the trend of the fluorescence intensity in different pH solutions. In addition to acidity, the effect of temperature on the fluorescence spectra of HSYA was also examined (Figure 2). As the temperature increased from 293K to 343K, the maximum fluorescence intensity is increased by almost 10 times. Thus, the fluorescence spectra of HSYA was sensitive to both acidity and temperature.

#### 3.1.2. Effects of Common Ions on HSYA Spectra

**Figure 3:**
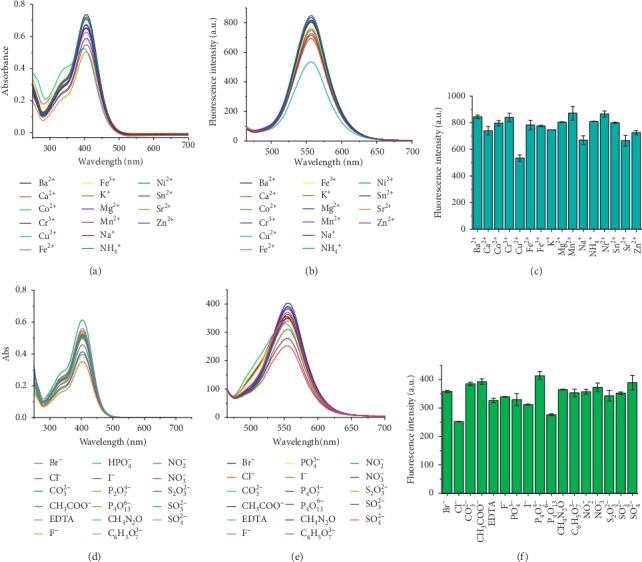
(a, d) Absorption spectra and (b, e) fluorescence spectra of HSYA in the presence of common cations and anions. (c, f) The maximum fluorescence intensity in the presence of various cations and anions. The cations included: Na^+^, K^+^, Mg^2+^, Ca^2+^, Fe^2+^, Fe^3+^, Cu^2+^, Zn^2+^, Co^2+^, Ni^2+^, Mn^2+^, NH_4_^+^, Sn^2+^, Sr^+^, Cr^3+^, and Ba^2+^. The anions included: F^−^, Cl^−^, Br^−^, I^−^, CO_3_^2−^, CH_3_COO^−^, EDTA, PO_4_^3−^, P_2_O_7_^4−^, P_4_O_13_^6−^, NO_2_^−^, NO_3_^−^, S_2_O_3_^2−^, SO_3_^2−^, SO_4_^2−^, CH_4_N_2_O, and C_6_H_5_O_7_^3−^. All spectra were measured in PBS (PO_4_^3−^ = 10 mM, pH = 7) solution at room temperature, with excitation at 450 nm.

The absorption and fluorescence spectra of HSYA in the presence of common cations and anions were measured (Figure 3). After the addition of metal and ammonia ions, HSYA showed weak fluctuations in absorbance (Figure 3(a)). However, a slight blueshift in wavelength and an obvious enhancement in the short-wavelength fluorescence intensity were observed upon the introduction of Cu^2+^. As shown in Figures 3(b) and 3(c), the fluorescence intensity of HSYA presented a clear decrease only in the coexistence of Cu^2+^ among various cations. These results indicated that there may be coordination interactions between HSYA and Cu^2+^, leading to fluorescence quenching. The introduction of various anions caused the HSYA absorbance to fluctuate within a limited range (Figure 3(d)). In the presence of anions, the fluorescence intensity of HSYA also displayed irregular fluctuations within a small range (Figures 3(e) and 3(f)). Generally, except Cu^2+^, the common ions had less interference with the absorption and fluorescence of HSYA.

#### 3.1.3. Effect of Viscosity on HSYA Spectra

**Figure 4:**
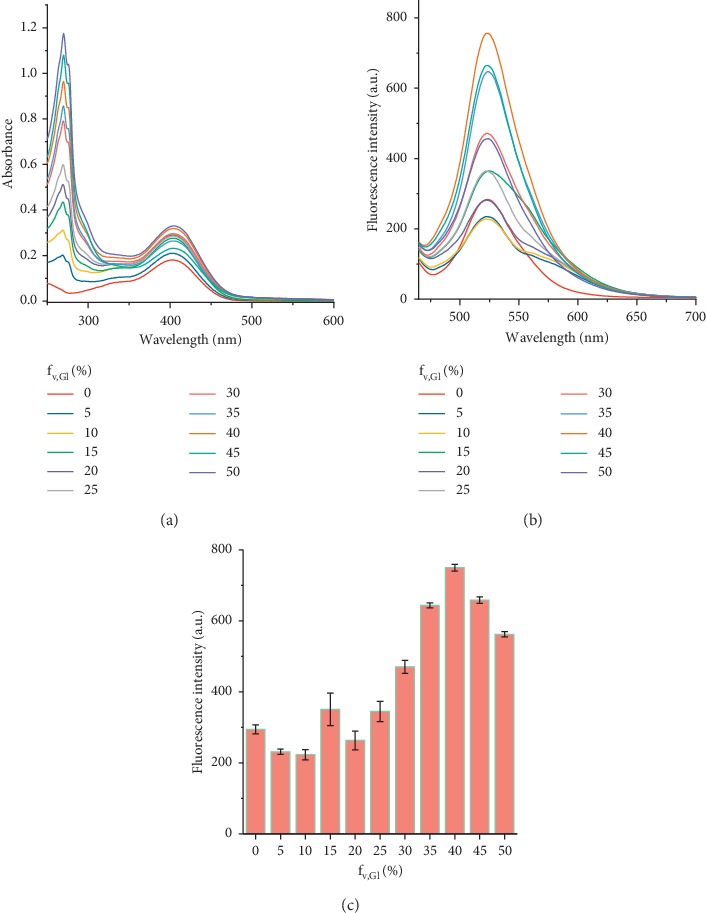
(a) Absorption spectra and (b) fluorescence spectra of HSYA in glycerol-water mixed solvents with an increasing volume fraction (0∼50%) of glycerol (f_w,Gl_). (c) Changes in maximum fluorescence intensity of HSYA with f_w,Gl_ in glycerol-water mixed solvents. All spectra were measured at room temperature, with excitation at 450 nm.

The effects of viscosity on the absorption and fluorescence spectra of HSYA were evaluated by dissolving HSYA in the glycerol-water mixed solvent, whose viscosity increased with increasing glycerol volume fraction (f_w_,_Gl_). As shown in Figure 4(a), the absorption spectra showed two bands below 300 nm and near 400 nm attributing to the absorption of glycerol and HSYA, respectively. The absorbance of HSYA at 400 nm generally increased with the promotion of f_w_,_Gl_. HSYA displayed maximum fluorescence emission centered at 525 nm in the glycerol-water mixed solvent (Figure 4(b)). As presented in Figure 4(c), the fluorescence intensity remained relatively stable under low-viscosity conditions (f_w_,_Gl_ < 25%) and then gradually upgraded with increasing f_w_,_Gl_ from 25% to 40%. This phenomenon resulted from that the increase in viscosity reduced energy consumption caused by intramolecular rotation of HSYA, thereby increasing the fluorescence quantum yield. The fluorescence intensity reached the highest level at 40% and then began to decrease with a further increase of f_w_,_Gl._ This result was expected due to that excessive glycerol might reduce the solubility of HSYA in the glycerol-water mixed solution. Therefore, the HSYA spectra were weakly disturbed in low-viscosity environment, but severely interfered under high-viscosity conditions.

#### 3.1.4. Effect of Surfactants and Borax on the Fluorescence Spectra of HSYA

**Figure 5:**
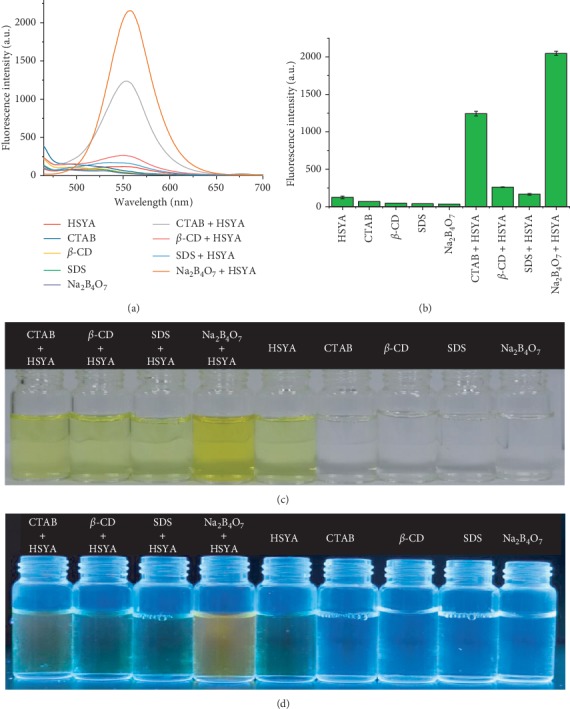
(a) The fluorescence spectra of HSYA in the presence of CTAB, *β*-CD, SDS, and Na_2_B_4_O_7_. All spectra were measured in PBS (PO_4_^3−^ = 10 mM, pH = 7) solution at room temperature, with excitation at 450 nm. (b) Changes in the maximum intensity of the fluorescence spectra shown in (a) at 550 nm. (c) Color images and (d) fluorescence images of the HSYA solution after the addition of surfactant and borax.

The fluorescence spectra of HSYA in the presence of CTAB, *β*-CD, SDS, and borax (Na_2_B_4_O_7_) were recorded and compared. HSYA molecules did not have a rigid planar configuration, so the absorbed energy was mainly consumed by intramolecular rotation, resulting in low fluorescence quantum yield. Therefore, HSYA emitted weak fluorescence under neutral conditions. After adding surfactants and borax, there was no significant shift in fluorescence wavelength (Figure 5(a)). However, with the introduction of borax, CTAB, *β*-CD, and SDS, the maximum fluorescence intensity increased by 16.6 times, 10.1 times, 2.1 times, and 1.3 times (Figure 5(b)), respectively. Considering that the added surfactants and borax had weak fluorescence in the detection system, their interference with the fluorescence measurement was very limited. Thus, it can be concluded that the increased fluorescence intensity was mainly due to the increase in the fluorescence quantum yield of HSYA. In the presence of borax, the HSYA solution appeared a deeper yellow color, while emitting bright orange-color fluorescence under UV irradiation (Figure 5(c)). These results revealed that borax had a significant sensitization effect on HSYA fluorescence.

### 3.2. Optimization on the Sensitization System of Borax-HSYA

#### 3.2.1. Optimum Concentration of Borax Used in the Borax-HSYA Sensitization System

**Figure 6:**
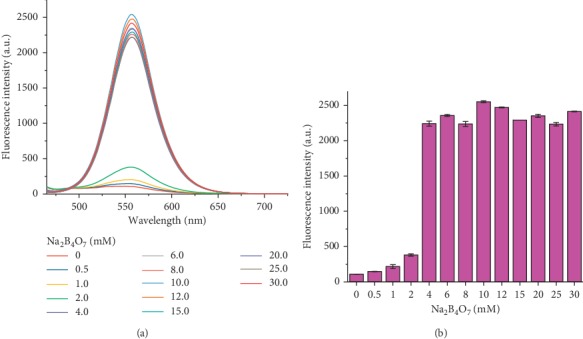
(a) Fluorescence spectra of HSYA in the presence of various concentrations (0∼30 mM) of borax. (b) Changes in maximum fluorescence intensity with borax concentrations. All spectra were measured in PBS (PO_4_^3−^ = 10 mM, pH = 7) solution at room temperature, with excitation at 450 nm.

As shown in Figure 6, with the increase of borax concentrations, the maximum fluorescence intensity was maintained at 555 nm, and no obvious wavelength shift was observed (Figure 6(a)). The maximum fluorescence intensity increased sharply 20-fold after addition of 4.0 mM boron and then reached a stable level when a higher borax concentration (4.0∼30.0 mM) was introduced (Figure 6(b)). To ensure the sensitization effect and avoid the negative effect of excessive boron on biological samples, a borax-HSYA sensitization system with an optimal concentration of 4.0 mM boron was utilized for further experiments.

#### 3.2.2. Linear Relationship between Fluorescence Intensity and HSYA Concentrations

**Figure 7:**
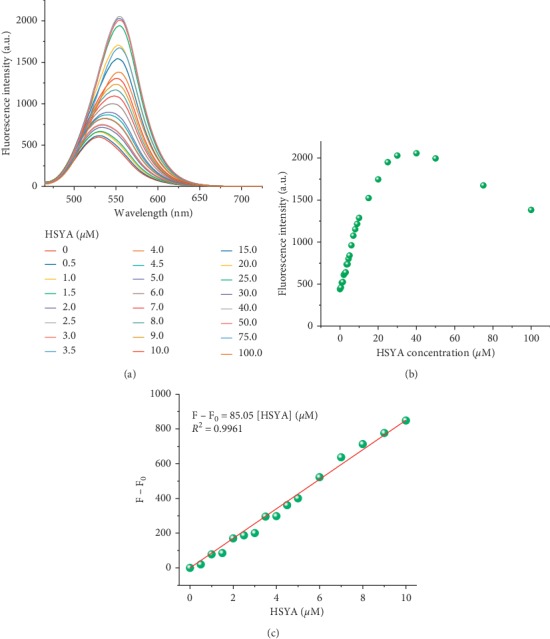
(a) Fluorescence spectra of HSYA at various concentrations (0∼1000 *μ*M) in the borax-HSYA sensitization system containing 4 mM borax. (b) The fluorescence intensity at 554 nm varied with HSYA concentrations ranging from 0 to 10 *μ*M (F_0_ and F are the fluorescence intensities before and after adding HSYA into the sensitization system). (c) Linear relationship between the increase in fluorescence intensity at 554 nm and HSYA concentration (0∼10 *μ*M). All spectra were measured in PBS (PO_4_^3−^ = 10 mM, pH = 7) solution at room temperature, with excitation at 450 nm.

In the optimized sensitization system containing 4 mM borax, the maximum emission wavelength was gradually red-shifted from 520 nm to 554 nm as the concentration of HSYA increased (Figure 7(a)). As the HSYA concentration increased from 0 to 40 *μ*M, the fluorescence intensity at 554 nm enhanced significantly and reached a maximum level at 40 *μ*M (Figure 7(b)). Then, further introduction of higher concentrations of HASY will result in a gradual decrease in fluorescence intensity. This phenomenon resulted from the aggregation-induced quenching effect, which was prevalent in all various organic dyes with *π*-conjugated system. As shown in Figure 7(c), the increase in fluorescence intensity at 554 nm and the HSYA concentration (0∼10 *μ*M) showed a good linear relationship (F–F_0_ = 85.05 × [HSYA] (*μ*M), *R*^2^ = 0.9926, where F_0_ and F are the fluorescence intensities before and after adding HSYA into the sensitization system, *R* is the linear correlation coefficient). The limit of detection (LOD) for the borax-HSYA sensitization system was calculated to be 8 nM according to the definition by IUPAC (LOD = 3*σ*/*k*, where *σ* is the standard deviation based on twenty times fluorescence scan of blank samples and *k* is the slope of the standard curve given in Figure 7(c)).

#### 3.2.3. Effect of Temperature and Light on the Stability of HSYA

**Figure 8:**
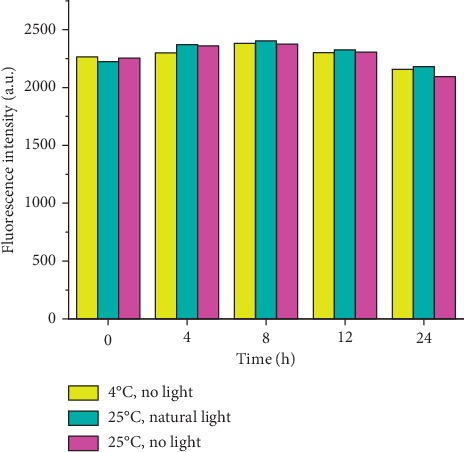
Time-dependent changes in the maximum fluorescence intensity of HSYA under different light and temperature conditions. The yellow, blue, and purple columns indicate samples stored in the dark at 4°C, in natural light at 25°C, and in the dark at 25°C, respectively. All spectra were measured in PBS (PO_4_^3−^ = 10 mM, pH = 7) solution containing 4 mM borax, with excitation at 450 nm.

As shown in Figure 8, in the absence of light interference, the maximum fluorescence intensity of HSYA in the borax-HSYA sensitization system remained almost unchanged at 4°C and 25°C for 24 hours. At the same time, under the same temperature conditions (25°C), the fluorescence intensity maintained a consistent level regardless of the presence of sunlight. The results showed that HSYA had good stability to light and low temperature (below 25°C) within 24 hours in the borax-HSYA sensitization system, which will be beneficial to the preservation and practical application of HSYA stock solution.

### 3.3. Toxicity Evaluation of the Borax-HSYA Sensitization System and Its Application for HSYA Imaging in Cells

#### 3.3.1. Cytotoxicity Evaluation

**Figure 9:**
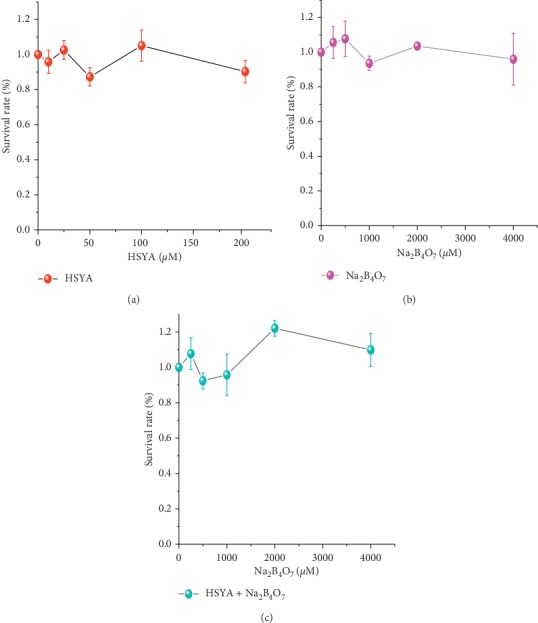
Survival rates of bladder cancer T24 cells after incubation with different concentrations of (a) HSYA (0∼200 *μ*M), (b) borax (0∼4000 *μ*M), and (c) borax (0∼4000 *μ*M) coexisting with HSYA (50 *μ*M), respectively.

Taking bladder cancer T24 cells as the example cell, the cytotoxicity of the borax-HSYA sensitization system was studied by measuring the survival rate using the CCK-8 method. After 24 hours of incubation with HSYA, borax, and the HSYA-borax mixture, respectively, the cell survival rate fluctuated between 90% and 110% (Figure 9), demonstrating that the borax-HSYA sensitization system has ignorable toxicity to T24 cells.

#### 3.3.2. Acute Toxicity Evaluation

**Figure 10:**
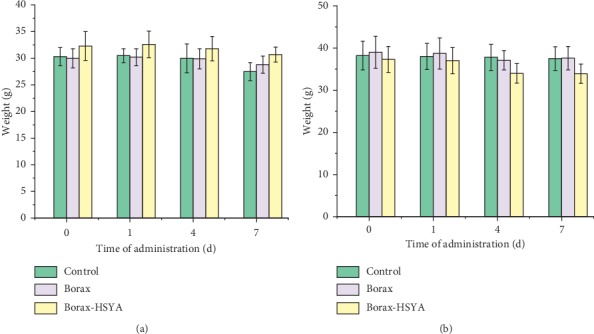
(a) Changes in the body weight of female mice and (b) male mice with experimental days. The green, purple, and yellow columns represent the group of mice injected with sterilized saline, borax, and the borax-HSYA mixture, respectively.

The effect of borax-HSYA injection on organisms was examined by acute toxicity test in mice. As shown in Figure 10, the mice injected with borax had almost the same weight as the mice in the control group. After injection of the borax-HSYA mixture, female mice had slightly increased weight, while male mice had slightly decreased weight. No mouse deaths and abnormal behavior were observed during the experiment. Therefore, when the concentrations of borax and HSYA were controlled within a certain range, the borax-HSYA sensitization system was expected to be safe to mice.

#### 3.3.3. Fluorescence Imaging of HSYA in Cells

**Figure 11:**
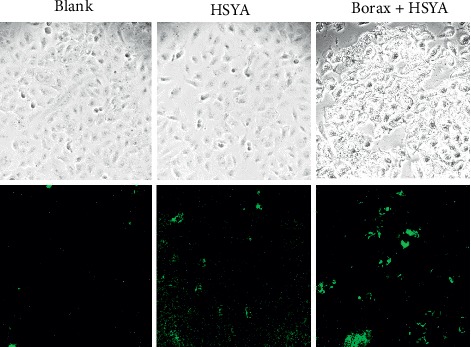
Fluorescence imaging of T24 cells without adding reagents (blank group), with adding only HSYA (HSYA group), and both HSYA and borax (borax + HSYA group). The top and bottom pictures show images of T24 cells in the bright and dark channels, respectively.

Based on the borax-HSYA sensitization system, the fluorescence imaging of HSYA in T24 cells was tested. As shown in Figure 11, the cells pretreated with only HSYA emitted weak fluorescence due to the low fluorescence quantum yield of HSYA. However, relatively strong fluorescence appeared in T24 cells incubated with HSAY and borax, suggesting that borax can enhance the fluorescence of HSYA even in a biological system.

## 4. Discussion

At present, the commonly used fluorescent microscopy chooses fluorescent dyes to label and localize intracellular molecules specifically. Organic fluorescent dyes are added to the culture medium to culture cells. After the cells absorb dyes, a series of metabolic processes are carried out to reach the corresponding sites. At present, fluorescent dyes used for fluorescence imaging labeling mainly include fluorescein [2], cyanine [3], rhodamine [4, 5], triphenylamine [6, 7], fluoroboron fluorescein [8]. But most of the organic small molecule fluorescent dyes have high cytotoxicity and poor biocompatibility, so cell imaging has limitations. Therefore, it is urgent to find appropriate fluorescent dyes, which generally require good liposolubility and water solubility, noncytotoxicity, normal cell metabolism, high intracellular stability, and a certain degree of fluorescence intensity.

HSYA is the main active ingredient in safflower, which has been proved to be effective in cerebral ischemia-reperfusion injury [9, 10]. However, its fluorescence intensity is not so obvious as that of other chemically synthesized fluorescent probes, and its stability is weak. Therefore, in order to better use HSYA in the treatment of diseases, it is necessary to make HSYA fluorescence. Strength enhancement and sensitization experiments were carried out, and appropriate sensitizers were selected to establish a high sensitivity sensitization analysis method.

The fluorescence intensity of HSYA decreases with the increase of pH. It is speculated that the reason is keto-enol tautomerism. HSYA is relatively sensitive to pH. With the increase of pH, the ketone form of HSYA will be transformed into enol structure form, and thus, the fluorescence intensity will change significantly, and the fluorescence color will change from yellow to orange-red. Because the enol form of HSYA has strong intermolecular hydrogen bonds, it is possible to produce larger aggregates in the form of HSYA ketone by enhancing self-aggregation, thus enhancing fluorescence. It can be seen that the spectral properties of HSYA are greatly affected by pH, and the fluorescence intensity remains stable under weak acidity and neutral conditions (4.0 < pH < 7.0). The fluorescence intensity of HSYA is also sensitive to temperature. Studies have found that curcumin fluorescence has dual sensitivity to temperature and pH value and is suitable as a fluorescent probe [11]. Therefore, the fluorescence intensity of HSYA is also sensitive to temperature and pH value. It can be considered that the fluorescence properties of HSYA can be combined with artificial conditions to modify the structure of the drug and research and application.

Through consulting the literature, we can see that there are some reasons for selecting borax for sensitization experiments. On the one hand, borax can protect spinal cord from injury and improve nerve function in the treatment of rat ischemia/reperfusion model [12]. This is similar to HSYA in the treatment of cerebral ischemia. On the other hand, a study tested the complexation of ellagic acid with borax and found that the interaction is insufficient for fluorescence when the concentration is below 0.05 mM, while the complex above 0.05 mM has fluorescence [13]. Therefore, borax is easy to form strong fluorescence complexes with some substances with special structures. In order to find suitable fluorescence sensitizer, borax is selected as the experimental reagent in this experiment to explore the sensitization or quenching effect of borax and its specific mechanism.

**Figure 12:**
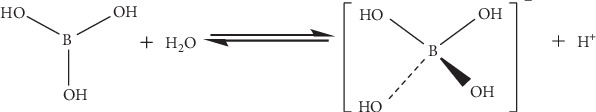
Chemical equation of hydrolysis of borax dissolved in water.

**Figure 13:**
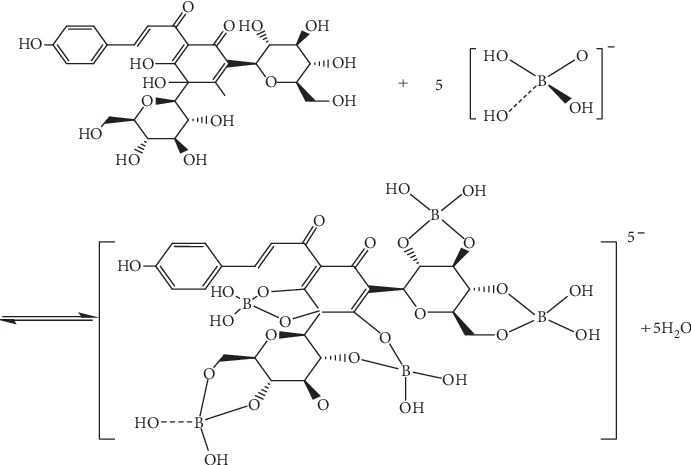
Chemical equation for the formation of large rigid planar structure between hydrolysate of borax and HSYA.

Ciprofloxacin (CIP) has fluorescence properties due to its conjugated system and rigid structure. The coordination of carbonyl group and carboxyl group with Tb^3+^ in the molecular structure sensitizes the fluorescence at 544 nm [14]. At the same time, we know that there may be the following hydrolysis processes in borax (sodium tetraborate decahydrate, Na_2_B_4_O_5_(OH)_4_·8H_2_O) solution. B_4_O_5_(OH)_4_^2−^ + 5H_2_O = 4H_3_BO_3_ + 2OH^−^, and the B in the generated H_3_BO_3_ is an electron-deficient atom, which binds to OH^−^ in water and then binds to every two adjacent OH^−^ in HSYA to form a sufficiently large and sufficient amount. Enough annular structure and rigid planar structure of the system increase the fluorescence intensity. The reaction formula is shown in Figures 12 and 13.

According to the principle of reaction between borax and HSYA, and considering that the fluorescence intensity of HSYA is greatly affected by pH and temperature, the influence of anion and cation is limited. Under neutral conditions, low viscosity, and no interference of Cu^2+^, HPO_4_^−^ and I^−^, the condition is close to the internal environment of organism, and then the fluorescence sensitization of borax-HSYA is preliminarily established. The analytical method is expected to apply the sensitizing system to the study of drug action *in vivo*.

This study showed that the borax-HSYA sensitization system had almost no toxic effect on bladder cancer T24 cells and mice within the range of 100 times the dosage of drug equivalent to normal intravenous drip. On the premise of nontoxicity to cells and organisms, the sensitization system does not rely on fluorescent molecular probes or related fluorescent dyes, but on the basis of self-fluorescence of drugs to a certain extent, and can perform cell fluorescence imaging of the sensitization system. The sensitization system can be further obtained by structural modification. Biofluorescent drugs with high sensitivity, nontoxicity, and good biocompatibility can explore the specific mechanism and metabolic pathways of drugs and organisms by means of drug fluorescence and drug metabolism.

Because some drugs have fluorescence, but the fluorescence intensity is weak, appropriate modification of drug structure can be considered to obtain the desired structure and properties, and a sensitization analysis system can be established based on the specific fluorescence properties. The purpose of this study is to provide some references for drug with fluorescence but weak fluorescence intensity to enhance its fluorescence intensity through fluorescence sensitization system, and for drug metabolic pathway research and related new drug research and development.

## Data Availability

The data analyzed for this study are available from the corresponding author upon reasonable request.

## Supplementary Materials

Supplementary Materials Supplementary material containing the mass spectrum and NMR spectrum of the compound HSYA.
