# Supplementary material for: Establishment of Fluorescence Sensitization Method for Hydroxysafflor Yellow A
Source: Evid Based Complement Alternat Med. 2020 Apr 27;2020:3027843. doi: 10.1155/2020/3027843 (PMC7204355; doi:10.1155/2020/3027843)

Mass spectrum

Agilent 1200 LC system & MS instrument:
API 4000 QTRAP Triple Quadrupole Mass Spectrometer with Electrospray Ionization Source (ESI)

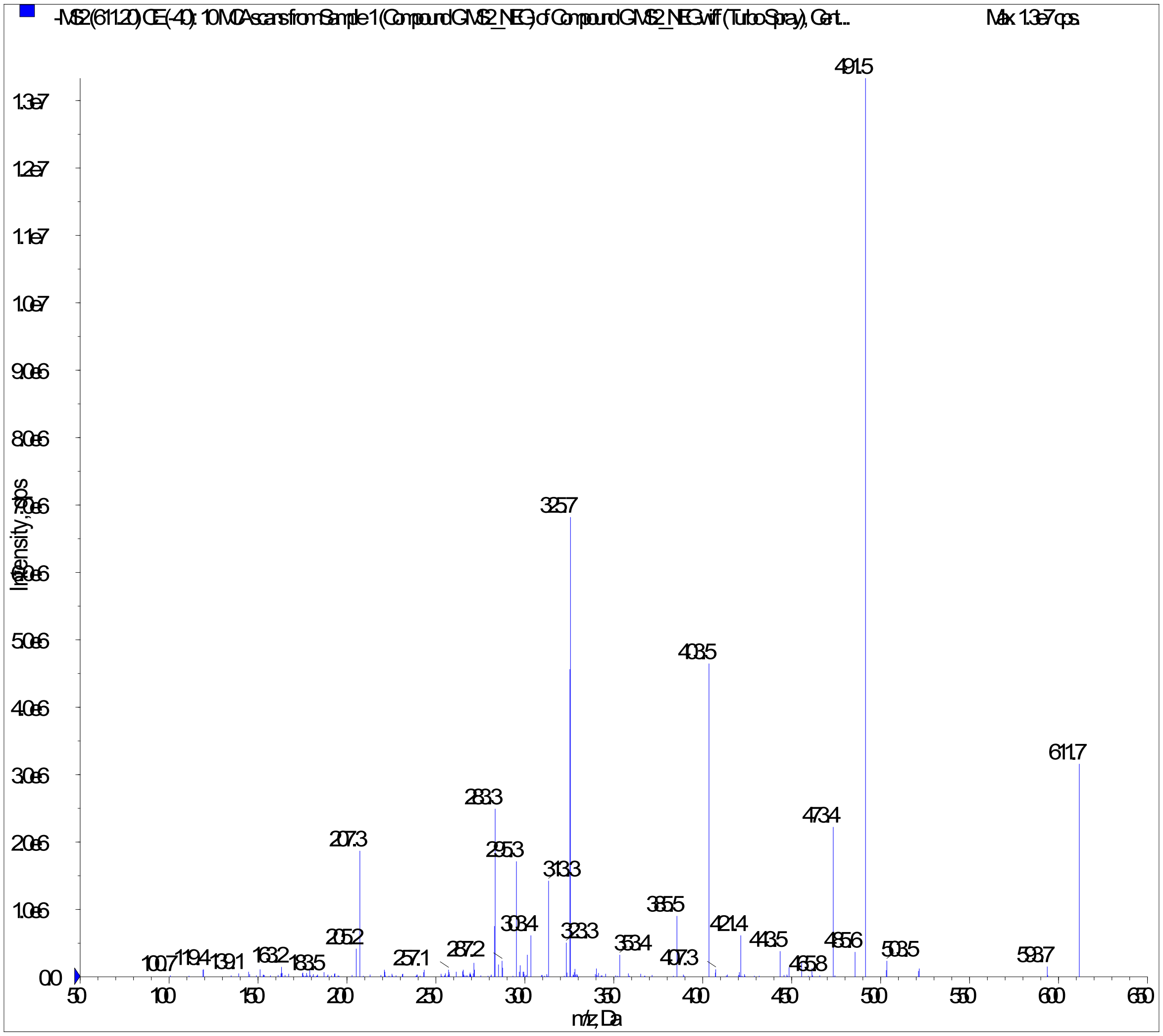

Molecular weight and secondary mass spectrum of compound HSYA

^{1}H-NMR spectrum and ^{13}C-NMR spectrum

Instrument model: BRUKER AVANCE Ⅲ 500 superconducting nuclear magnetic resonance instrument
Test conditions: solvent: DMSO, internal standard: TMS

^{1}H-NMR spectrum

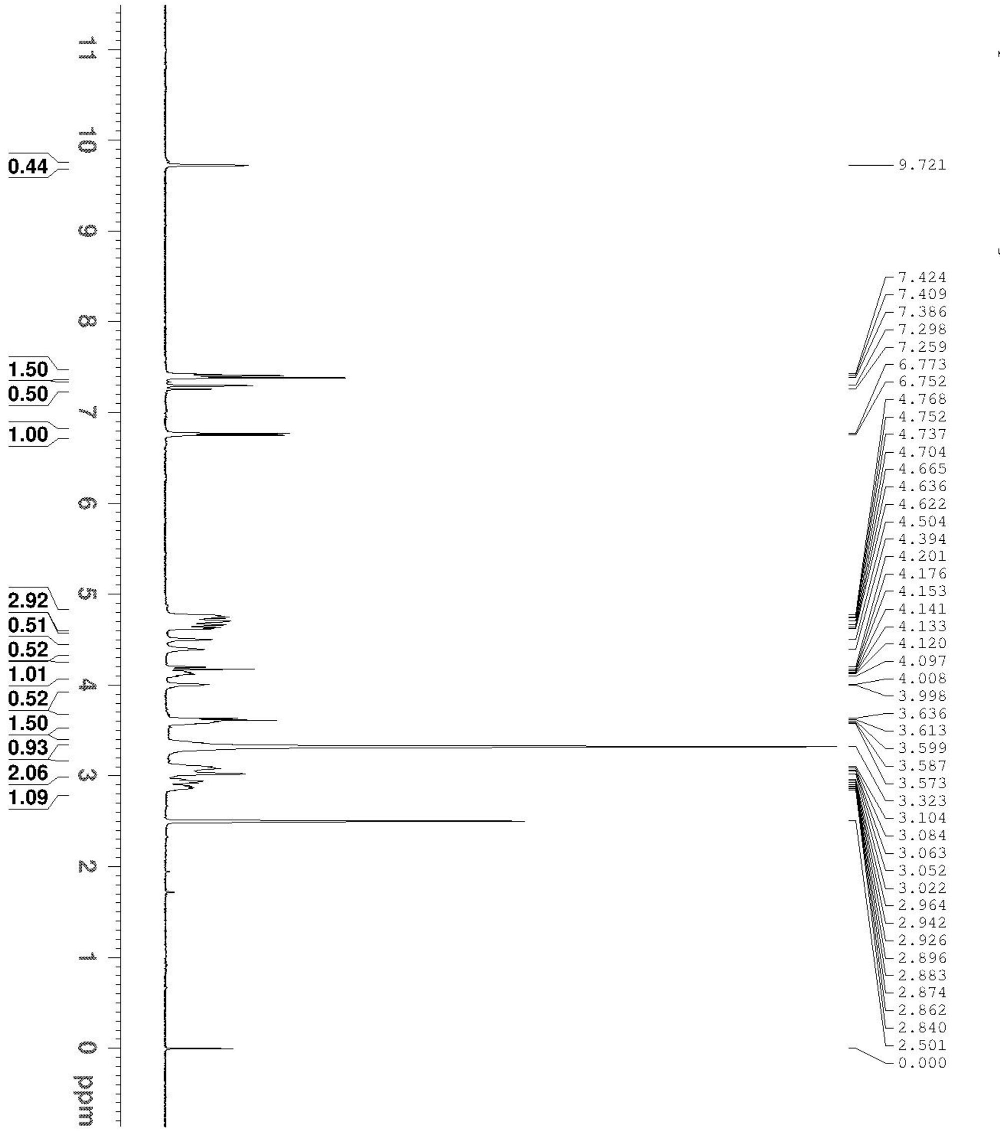

^{13}C-NMR spectrum

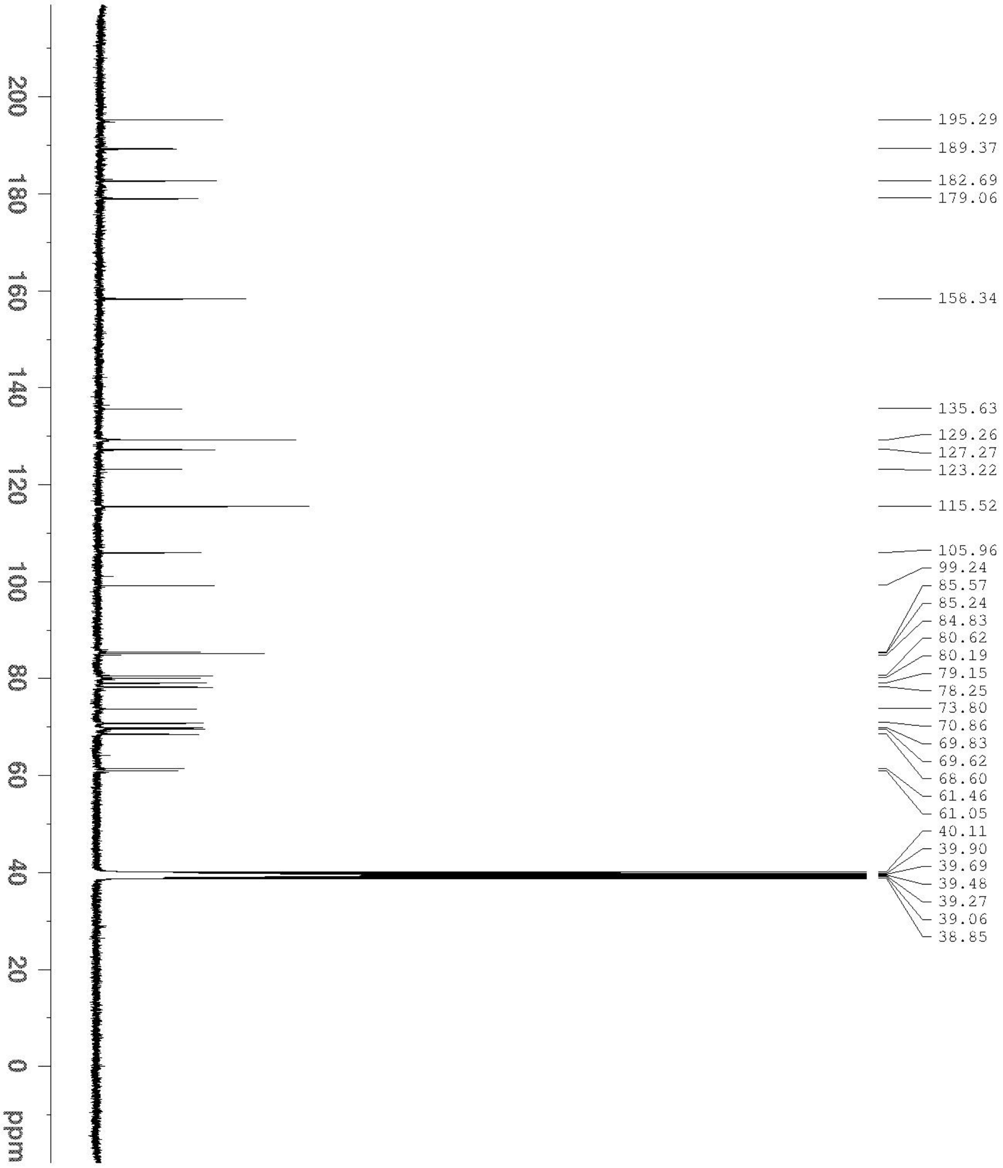

Supplement: Supplementary Materials — Supplementary material containing the mass spectrum and NMR spectrum of the compound HSYA. [file 3027843.f1.pdf]
